# Mechanistic Study of Tetrahydrofuran- acetogenins In Triggering Endoplasmic Reticulum Stress Response-apotoposis in Human Nasopharyngeal Carcinoma

**DOI:** 10.1038/srep39251

**Published:** 2016-12-21

**Authors:** Shin-Hun Juang, Chang-Ying Chiang, Fong-Pin Liang, Hsiu-Hui Chan, Jai-Sing Yang, Shih-Hao Wang, Yu-Chin Lin, Ping-Chung Kuo, Meng-Ru Shen, Tran Dinh Thang, Bui Thi Minh Nguyet, Sheng-Chu Kuo, Tian-Shung Wu

**Affiliations:** 1 Department of Pharmacy, Tajen University, Pingtung 907, Taiwan; 2 Department of Medical Research, China Medical University Hospital, China Medical University, Taichung 404, Taiwan; 3 School of Pharmacy, China Medical University, Taichung 404, Taiwan; 4 Department of Pharmacy, National Cheng Kung University, Tainan 701, Taiwan; 5 Department of Pharmacology, Obstetrics & Gynecology, National Cheng Kung University, Tainan 701, Taiwan; 6 Department of Chemistry, Vinh University, Vinh City, Nghean province, Vietnam

## Abstract

For past three decades, numerous studies have elucidated the antiproliferative effects of acetogenins in hopes of developing a new class of clinical anticancer agents. However, clear and definitive action mechanisms of acetogenins were less clarified. In the present study, three tetrahydrofuran (THF)-containing acetogenins were found to have potent and selective antiproliferative activity against human nasopharyngeal carcinoma (NPC) cell lines and their methotrexate-resistant counterparts. The THF-containing acetogenins induced G_2_/M phase arrest, mitochondrial damage and apoptosis, and increased cytosolic and mitochondrial Ca^2+^ in NPCs. Microarray analysis of NPC-TW01 cells treated with squamostatin A, a non-adjacent bis-THF acetogenin, demonstrated an increased endoplasmic reticulum (ER)-stress response (ESR). Enhanced ESR in squamostatin A-treated cells was confirmed by real-time PCR, Western blot and shRNA gene knockdown experiments. Although our results showed that squamostatin A-induced ESR was independent of extracellular Ca^2+^, the presence of extracellular Ca^2+^ enhanced the antiproliferative effect of acetogenins. *In vivo* analyses demonstrated that squamostatin A showed good pharmacokinetic properties and significantly retarded NPC tumor growth in the xenograft mouse model. Conclusively, our work demonstrates that acetogenins are effective and selective inducers of the ESR that can block NPC proliferation, and illustrate a previously unappreciated antitumor mechanism of acetogenins that is effective against nasopharyngeal malignancies.

The incident of nasopharyngeal carcinoma (NPC) is extremely prevalent in South-East Asia, particular in Guangdong province of China (25 cases per 100,000 people)^1^ and also is the tenth leading cause of mortality among male cancer patients in Taiwan. Due to the anatomical location of the nasopharynx, early detection of NPC during routine physical exams has proven to be very difficult. Consequently, 20% of all NPC patients have distant metastases in the bone, lung, mediastinum and liver at the time of diagnosis^2^. As a result, the five-year survival rate of NPC patients is between 10 and 40%^3^. Although many clinical trials have shown that patients receiving pre-radiation chemotherapy with methotrexate (MTX), cisplatin and 5-fluorouracil could significantly improve the five-year survival rate of metastatic NPC patients^4^,^5^, a considerable number of NPC patients develop drug-resistance and succumb to NPC as a result of disease progression. Therefore, new and effective treatments for NPC patients are urgently needed. Because the incidence of NPC in Western societies is relatively low and the development of new therapeutics for NPC has not been a high priority for many pharmaceutical institutions, the discovery of new pharmaceutical agents targeting NPC has been a high priority for the scientific community and governmental health agencies in South-East Asia for many years.

In the past 30 years, the scientific field has successfully identified numerous useful chemicals from natural sources for the treatment of diseases^6^. *Annonaceous* acetogenins (ACGs) are compounds found exclusively in *Annonaceous* plants in tropical and subtropical regions of South-East Asia. Most *Annonaceous* acetogenins are characterized by unique C_32_ or C_34_ unbranched fatty acids with a single, adjacent or nonadjacent tetrahydrofuran (THF) or tetrahydropyran (THP), oxygen-bearing moieties and a β-lactone at the end of fatty acid chain^7^. In 1982, uvaricin was identified as the first ACG compound and contained potent anti-tumor activity with an IC_50_ in the nanomolar range. ACG derivatives have since become promising new pharmaceutical candidates for treating various cancers^8^,^9^,^10^ and chemo-resistant malignancies^11^. However, after more than three decades of intensive research, with over 400 natural and synthetic ACGs tested^12^, the mechanism of action remains largely elusive. Several molecular pathways have been proposed, such as disruption of mitochondrial complex I^13^, the generation of superoxide anion and hydrogen peroxide^14^, decreases in both cAMP and cGMP levels^15^, the induction of cell-cycle arrest^16^,^17^ or apoptotic cell death induced by elevated cytosolic Ca^2+ ^18. However, none of these mechanisms are able to fully explain the anti-tumor properties observed of ACGs^19^. The lack of a defined biological mechanism of action has greatly hindered the usage of ACGs as antitumor agents in the clinic.

The endoplasmic reticulum (ER) is involved in the folding and post-translational modification of secretory and membrane-bound proteins, lipid biosynthesis and intracellular calcium homeostasis^20^,^21^ and is crucial for normal cellular function and survival. In addition to the biosynthetic capacity, the ER is the highest concentrations calcium containing organelle in the cell and sequestered calcium can be released in response to secondary messengers, protein kinases and other modulators^22^,^23^. Multiple disturbances, including nutrient deprivation, hypoxia, redox stimulation and disturbances of calcium flux, lead to the accumulation of unfolded and/or misfolded proteins in the ER lumen, causing ER-stress responses (ESR)^24^. ESR triggers the unfolded protein response (UPR), a unique cytoprotective signaling cascade^25^ that is intended to re-establish homeostasis and normal ER function by inhibiting mRNA translation but inducing the expression of genes that are capable of enhancing protein folding capacity and ER-assisted degradation^26^. However, highly accumulation of unfolded and/or misfolded proteins in the ER has been associated with a wide range of diseases, including neurodegeneration, stroke, cardiac disease, diabetes, muscle degeneration and cancer^27^. Although, several lines of evidence suggests that the ESR supports tumor cell survival under adverse conditions^28^, prolonged ER stress ultimately results in apoptosis and cell death^29^. Therefore, a number of ESR-induce drugs including Celecoxib and its analogs^30^,^31^, Bortezomib^29^,^32^,^33^ and Nelfinavir^34^, are currently under clinical trials or development of new cancer chemotherapy agents.

In order to identify the potent anti-proliferative agents against NPC, hundreds of compounds were evaluated against two human NPC cell lines (NPC-TW01^35^ and HONE-1^36^).The results showed that several ACG compounds could effectively inhibit the growth of NPC cells with an IC_50_ in the nanomolar range and could effectively overcome methotrexate resistance. Furthermore, our results showed that all three categories of THF-containing acetogenins (THF-ACGs) could induce similar phenotypic changes, including G_2_/M cell cycle-arrest, increased cytosolic and mitochondrial Ca^2+^ levels, mitochondrial damage and apoptosis. Microarray analysis data of squamostatin A-treated NPC-TW01 cells suggested that THF-ACG may augment the activation of ESR and was later confirmed by real-time PCR, Western blot analysis and siRNA knockdown experiments. Contrast to a previous report^18^, our study showed that the ERS response induced by THF-ACG was independent of extracellular Ca^2+^, although the presence of extracellular Ca^2+^ enhanced the antiproliferative effect of ACGs. *In vivo* experiments showed that squamostatin A has a good pharmacokinetic profile and anticancer properties against NPC growth in the s.c. xenograft murine experiments. Collectively, our findings suggest that THF-ACGs act as effective and selective inducers of the ESR, blocking NPC proliferation and illustrating a previously unappreciated antitumor mechanism that is effective against nasopharyngeal malignancies.

## Results

### THF-ACGs show potent and selective growth inhibition toward parental and MTX-resistant NPC cells

To evaluate the growth inhibitory effect, three class of THF-ACGS, non-adjacent bis-THF (Squamostatin A), adjacent bis-THF (squamocin M) and mono-tetrahydrofuran (corossolone), were tested against cancer cell lines of different origin. Although different classes of THF-ACGs demonstrated varying levels of antiproliferative potency toward several tumor types, all three THF-ACGs showed potent growth inhibitory activity toward both parental as well the MTX-resistant NPC-TW01 and HONE-1 cells with IC_50_ values in the nanomolar range (Table 1). These results suggested these THF-ACGs compounds are potent antiproliferative agents against NPC cells and can overcome the methotrexate-induced resistance. Interestingly, several oral carcinoma cell lines, which are anatomically close to the nasopharynx, nasopharynx carcinomas including FaDu cells, were insensitive to THF-ACGs treatment (Supplemental Table 1). Moreover, the IC50 of squamostatin A toward the normal fibroblast cell, Detroit 551, was found at least 50 folds higher than that of NPC cells, implying a lowered adverse effects if squamostatin A were used in an *in vivo* test.

### THF-ACGs induce cell-cycle arrest in G_2_/M, cytosolic and mitochondria Ca^2+^ accumulation and apoptosis

To further investigate the mechanisms underlying the antiproliferative effect of THF-ACGs, THF-ACGs-treated tumor cell lines were subjected to cell-cycle distribution and apoptosis analysis by flow cytometry. Cell cycle analysis showed that all three THF-ACGs compounds induced G_2_/M accumulation (Table 2). Furthermore, Annexin-V analysis showed that the apoptotic cell population increased from 3% to 14~19% after cells were co-cultured with THF-ACGs for 36 hours (Table 3). The above results suggested that the THF-ACGs treatment could induce NPC cells arrest at G2/M and cause cell death through the apoptotic pathway.

The previous report by Liaw *et al*. suggested that ACG treatment induces a dramatic increase in cytosolic Ca^2+^, resulting in tumor cell death^18^. Our results also showed that cytosolic Ca^2+^ levels were elevated by 50~120% after treatment with all three THF-ACGs. Interestingly, a ~30% increase in mitochondrial Ca^2+^ levels were observed, which was not reported by Liaw *et al*. (Table 4). Recent studies have suggested that mitochondrial Ca^2+^ elevation could induce mitochondrial disruption and apoptotic cell death^37^, therefore, the mitochondrial membrane potential of THF-ACG-treated NPC-TW01 cells were measured by DiOC_6_ staining. Results showed a 27% to 37% drops in mitochondrial membrane potential in NPC-TW01 cells co-cultured with THF-ACGs for 24 hours (Table 4).

The above results demonstrate that despite the structure difference of these THF-ACGs, all three molecules induced similar phenotypic changes, including G_2_/M cell-cycle arrest, increased cytosolic and mitochondrial Ca^2+^, mitochondrial damage and apoptosis, suggesting that all three THF-ACGs might act through a similar antiproliferative pathway to block NPC tumor growth.

### Squamostatin A induces ER stress in NPC-TW01 cells

To further investigate the antiproliferative mechanisms of THF-ACGs, squamostatin A, a highly potent ACG that blocks tumor growth but showed limited toxicity toward normal Detroit 551 cells (Table 1), was chosen for further mechanistic study. To understand the change of gene expression profile following THF-ACGs treatment, total RNAs were isolated at different time points (18 and 36 hours) and the dose of squamostatin A (6 and 12 nM) treated NPC-TW01 cells and subjected to cDNA microarray analysis. Compared to untreated NPC-TW01, the microarray analysis data showed 84 up-regulated and 85 down-regulated genes (data not shown). The analysis of canonical pathway maps, representing a set of approximately 650 signaling and metabolic maps, indicated that squamostatin A might induce ESR (Supplemental Fig. 1A). Using GeneGo software, we identified 17 ER-stress-related genes that showed at least a two-fold change in expression (12 up-regulated and 5 down-regulated; Supplemental Fig. 1B).

**Figure 1:**
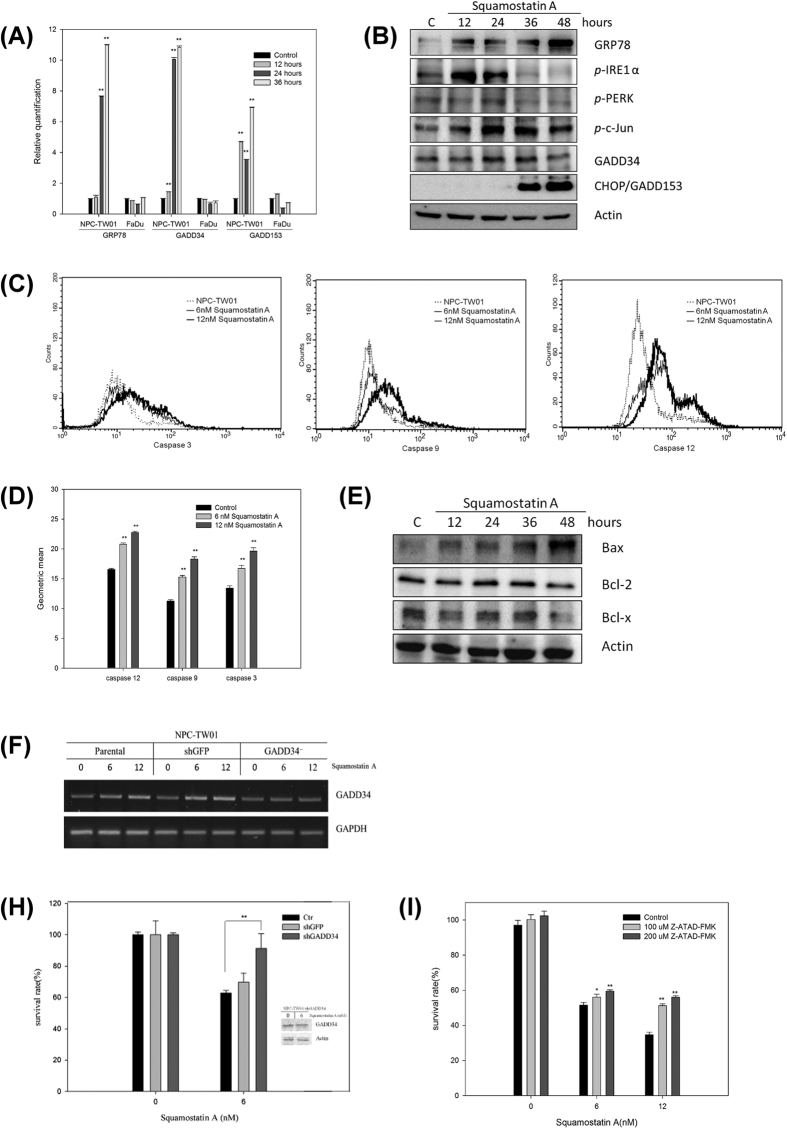
Effects of squamostatin A on the ER-stress response. (**A**) The mRNA levels of ER-stress-related genes. NPC-TW01 and FaDu cells were treated with the IC_50_ concentration of squamostatin A for the indicated times. The mRNA levels of GRP78, GADD34 and GADD153 were evaluated using qPCR with GAPDH as an internal control. The control group indicates vehicle-treated cells. (**B**) The protein levels of ER-stress-related proteins. NPC-TW01 cells were treated with 6 nM of squamostatin A for the indicated times. The protein levels and phosphorylation statuses of ER-stress-related proteins were evaluated using western blot analysis with β-actin as an internal control. The control group indicates vehicle-treated cells at time zero. (**C**) Flow cytometric analysis of caspase-12, -9 and -3 activities in NPC-TW01 cells treated with various concentrations of squamostatin A. (**D**) The activities of caspase-12, -9 and -3 were measured using an assay for the specific cleavage of fluorogenic tetrapeptides, and the fluorogenic intensities are shown as the GEO mean values. The control group indicates vehicle-treated cells. (**E**) Western blot analysis of apoptosis-related proteins. NPC-TW01 cells were treated with 6 nM of squamostatin A for the indicated times. The protein levels of mitochondrial apoptotic proteins were evaluated using western blot analysis with β-actin as an internal control. The control group indicates vehicle-treated cells at time zero. (**F**) The mRNA level of GADD34 in transient transfected cell lines. After treated with squamostatin A for 24 hours, the transient transfected shGADD34 NPC-TW01 cells were harvested for PT-PCR analysis. (**G**) GADD34 knockdown effect. The cell survival rate of transient transfected shGADD34 NPC-TW01 cells was measured after treatment with squamostatin A for 48 hours. The insert figure is the GADD34 protein expression levels in shGADD34 transfected cells without or with squamostatin A treatment. (**H**) Caspase-12 inhibitory effect. Before treatment with different concentrations of squamostatin A, cells were pretreated with different concentrations of a caspase 12-specific inhibitor (Z-ATAD-FMK) for 1 hour, and cell survival was measured after treatment with squamostatin A for 72 hours. The control group indicates vehicle-treated cells. *refer to *P* < 0.05, **refer to *P* < 0.01 by Student’s t-test. All error bars represent the standard deviations.

To verify the microarray results, the expression level of several ESR-relative proteins was analyzed by RT-qPCR and Western blots. The mRNA expression of Grp78, GADD34 and CHOP/GADD153 were up-regulated in squamostatin A-treated NPC-TW01 cells. No change in Grp78, GADD34 or CHOP/GADD153 was detected in the squamostatin A-treated FaDu cells, which are insensitive to ACGs (Fig. 1A). These results strongly indicate that the up-regulation of ESR genes contributes to the antiproliferative effect of squamostatin A. Furthermore, the up-regulation of Grp78 protein was found 12 hours after treatment, but significant induction of CHOP/GADD153 was not observed until 36 hours after treatment. The phosphorylation of Ire-1 and c-Jun was found at 12 hours after squamostatin A treatment and weak activation of PERK was also observed at 24 hours after treatment in NPC-TW01 cells (Fig. 1B). Because activation of caspase-12 is a typical biomarker^38^,^39^,^40^ for ESR in human NPC cells and the trigger of the mitochondrial-associated caspase cascade. the activity of caspase-12 and the mitochondrial apoptotic caspases, caspase-9 and -3, was measured in squamostatin A-treated NPC-TW01 cells by flow cytometry. The data showed that squamostatin A could induce the activity of caspase-12, -9 and -3 in a concentration-dependent manner after 36 hours of incubation (Fig. 1C and D). However, no alteration in caspase-8 activity was observed until 60 hours after drug treatment (data not shown). Furthermore, up-regulated of Bax and down-regulated of Bcl-xL in squamostatin A treated NPC-TW01 cells was found, further indicating the apoptotic stage of these NPC cells (Fig. 1E).

To establish whether drug-induced ER stress and cellular apoptosis were correlative or only causally related, GADD34 expression, the common factor for all three ER stress signal transduction pathways^41^ in the NPC-TW01 was knocked down by GADD34-shRNA. The shGFP (green fluorescent protein) transfected NPC-TW01 was established and used as a control. Both shGADD34 and shGFP-transfected cells were treated with squamostatin A and the mRNA level of GADD34 and cell surviving rate were determined. The up-regulated GADD34 mRNA level were found in squamosatin A-treated parental and shGFP transfected cells, but squamastain A upregulated GADD34 mRNA expression could be blocked when cells were transfected with shGADD34 (Fig. 1F). Although the cell survival was significantly (p < 0.05) increased in the GADD34 shRNA transfected cells but did not abrogate the squamostatin A cytotoxicity toward the cells. This result might be due to knock-out efficiency of GADD34 shRNAs were used. The similar blockage efficiency of squamastain A-induction of GADD34 protein was found in the shGADD34s transfected cell (Fig. 1G insert).

Furthermore, inactivation of caspase-12 activity by pretreatment with a caspase-12-specific inhibitor (Z-ATAD-FMK) could also significantly block the toxicity of squamostatin A against NPC-TW01 cell growth (Fig. 1H). These results suggest that the induction of ESR might play a crucial role in squamostatin A-induced apoptotic cell death.

### Squamostatin A induces ER damage, ER Ca^2+^ release into the cytosol and causes cytosolic Ca^2+^ accumulation

**Figure 2:**
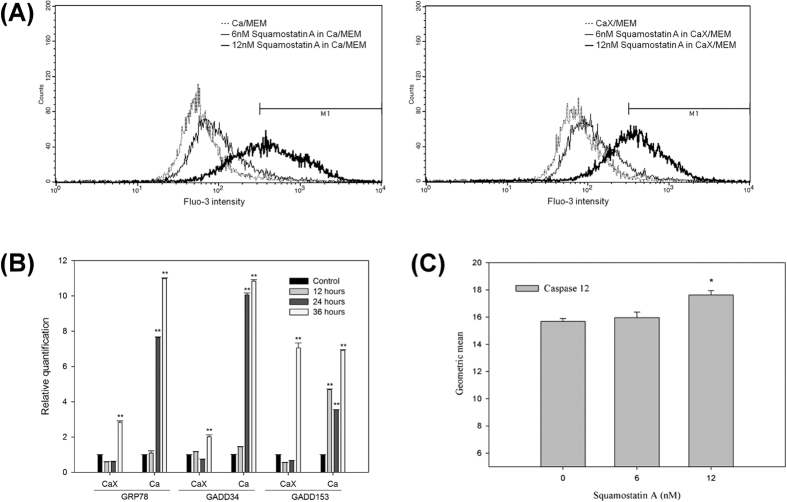
Investigation of the role of extracellular calcium on the squamostatin-A-mediated ER stress response in NPC-TW01 cells. (**A**) Cells were treated with various concentrations of squamostatin A in either complete medium (Ca/MEM) (*top panel*) or calcium-free medium (CaX/MEM) (*bottom panel*) for 24 hours. Cytosolic calcium concentrations were measured using the calcium indicator dye Fluo-3. (**B**) Cells were treated with the IC_50_ concentration of squamostatin A in calcium-free medium (CaX/MEM) for the indicated times. The mRNA levels of GRP78, GADD34 and GADD153 were evaluated using qPCR with GAPDH as an internal control. (**C**) Cells were treated with various concentrations of squamostatin A in calcium-free medium (CaX/MEM) for 24 hours. Caspase 12 activity was measured using an assay for the specific cleavage of fluorogenic tetrapeptides. *refer to *P* < 0.05.

Previously, Liaw *et al*. hypothesized that ACGs could chelate and transport Ca^2+^ from the extracellular culture medium across the cell membrane, leading to increased cytosolic Ca^2+^ levels and ultimately resulting in cell death^18^. To investigate the importance of extracellular Ca^2+^, NPC-TW01 cells were cultured in Ca^2+^-free medium for 24 hours, then treated with squamostatin A, and the change in the IC_50_ values for squamostatin A against NPC-TW01 cell growth, cytosolic Ca^2+^ concentrations, the expression of ESR-related genes and caspase-12 activity were measured. To our surprise, the sensitivity of NPC-TW01 cells to squamostatin A in Ca^2+^ and Ca^2+^-free media was very similar, (6 nM and 8 nM, respectively). Although the cytosolic Ca^2+^ levels in the two culture conditions (media with and without Ca^2+^) were very similar (Fig. 2A), the induction of ESR-related genes of squamostatin A-treated NPC-TW01 occurred 12 hours later in the Ca^2+^-free media, and the induction of these genes was weaker (Fig. 2B). Although the ability of squamostatin A to induce the activation of caspase-12 was not affected, the maximal induction of caspase-12 activation in the Ca^2+^-free media also occurred 12 hours later than the cells cultured in the Ca^2+^ media (Fig. 2C). The 12 nM squamostatin A treatment group was significant (*p* < 0.05) compared to untreated group. These results suggest that the increase in cytosolic Ca^2+^ levels induced by squamostatin A treatment is due to the induction of the ESR and Ca^+2^ release from the ER; however, extracellular Ca^2+^ may also augment the antiproliferative effect of squamostatin A.

### Squamostatin A is involved in the regulation of calcium signaling

**Figure 3:**
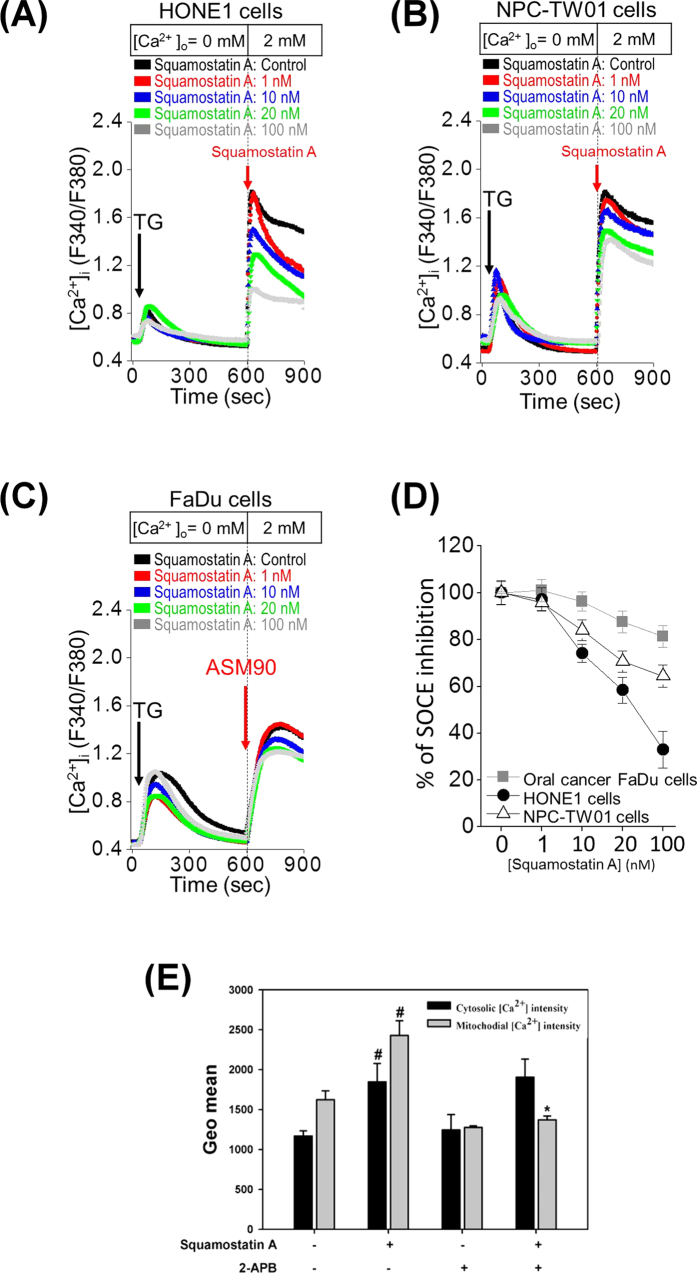
Squamostatin A inhibits the activation of the store-operated Ca^2+^ entry (SOCE). **(A,B,C)** Mean traces for the measurement of intracellular Ca^2+^ concentration ([Ca^2+^]_i_) in three different cancer cell lines. Each trace is the mean value from at least 60 different cells. The SOCE amplitude indicates the rise of [Ca^2+^]_i_ in replenishment of [Ca^2+^]_o_ from 0 to 2 mmol/L. Arrow, adding 2 μM thapsigargin or different concentrations of squamostatin. **(D)** Dose-response curves for squamostatin A in blocking SOCE activation in HONE1, NPC-TW01 and FaDu cell lines. Each point in the dose–response curve represents mean ± SEM from at least 3 different experiments. (E) NPC-TW01 cells were treated with the IC_50_ concentration of squamostatin A with/without 2-APB (10 μM) for 24 hours, and the cytosolic and mitochondria calcium concentrations of the cells were measured by the calcium indicator dyes, Fluo-3 and Rhod-2, respectively. The fluorogenic intensities are shown as the GEO mean values. ^#^The squamostatin A treatment versus the non-treatment. *The squamostatin A treatment versus the squamostatin A and 2-APB co-treatment.

The store-operated Ca^2+^ entry (SOCE) is a major mechanism to regulate Ca^2+^ homeostasis in most types of epithelial cells. The dysregulation of Ca^2+^ homeostasis has been suggested as an important event in driving the expression of the malignant phenotypes, such as proliferation, migration, invasion, and metastasis^42^. To inhibit the activation of SOCE has been proposed as a strategy to inhibit tumor growth and metastasis^43^,^44^,^45^. Here, we used three different cancer cell lines to study whether squamostatin A affects the SOCE (Store-operated Ca^2+^ entry) activation. As shown in Fig. 3A–C, thapsigargin (TG), a SERCA (sarco/endoplasmic reticulum Ca^2+^ ATPase) pump inhibitor, blocked Ca^2+^ refilling into ER and led to ER Ca^2+^ depletion. SOCE was activated after replenishment of 2 mM Ca^2+^ buffer. When [Ca^2+^]i was replenished, two nasopharyngeal cancer HONE1 and NPC-TW01 cancer cells displayed a significant activation of SOCE, compared to oral cancer FaDu cells (Fig. 3A–C). In addition, squamostatin A dose-dependently inhibited SOCE activity in HONE1 and NPC-TW01 cells. On the other hand, FaDu cells were less sensitive to the squamostatin A treatment (Fig. 3D). For example, 100 nM squamostatin A inhibited SOCE activity by 20, 35 and 65% in FaDu, NPC-TW01 and HONE1 cells, respectively. These results reveal that squamostatin A differentially inhibits the SOCE activity in those cancer cell lines.

To further elucidate the calcium traveling in squamostatin A-treated NPC-TW01 cancer cells, 2-Aminoethoxydiphenyl borate (2-APB) was utilized to block InsP3 receptors or hinder the mitochondrial calcium uptake^46^. As shown in Fig. 3E, squamostatin A treatment in the presence of 2-APB could diminish the content of mitochondrial calcium, indicating the calcium in mitochondria comes from released calcium from ER or influx calcium form outside of cells after squamostatin A treatment.

### Squamostatin A exerts a good PK profile with potent tumor growth inhibitory activity *in vivo*

**Figure 4:**
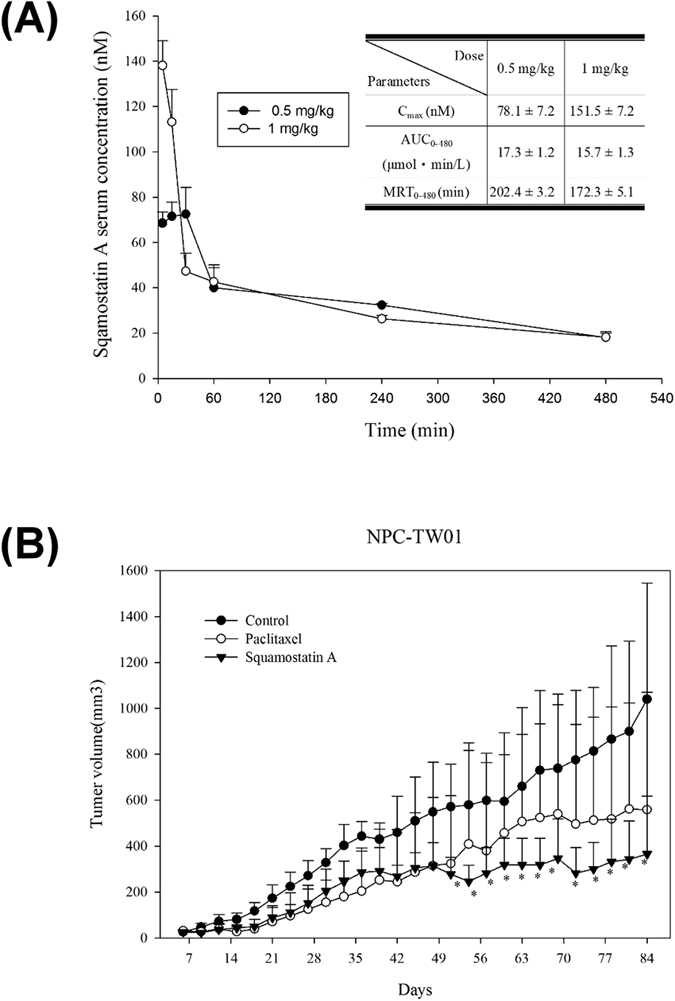
*In vivo* bioactivity of squamostatin A. (**A**) Serum concentrations and pharmacokinetic profiling (figure insert) of squamostatin A (0.5 mg/kg, n = 4, and 1 mg/kg, n = 6) after a single intraperitoneal injection. (**B**) *In vivo* antitumor activity of squamostatin A in a human NPC-TW01 xenograft model. NPC-TW01 cells were subcutaneously implanted in nude mice. After 7 days, the mice received *i.p.* administration of 0.5 mg/kg squamostatin A once every three days. Paclitaxel was used as a positive control at a dose of 10 mg/kg once a week. Tumor size was measured every three days. *P < 0.05 by Student’s t-test. All error bars represent the standard deviations.

Finally, we examined the pharmacokinetic properties, *in vivo* bioactivity and potential clinical utility of squamostatin A in mice. For PK studies, 0.5 and 1 mg/kg of squamostatin A were administered via intraperitoneal injection into BALB/c mice. Squamostatin A levels in blood samples were then measured by LC/MS/MS. Importantly, the PK data showed that the concentration of squamostatin A in the serum was over 20 nM over the 8-hour testing period in both 1 mg/kg and 0.5 mg/kg dose groups; a nearly 2- to 3-fold higher than the *in vitro* IC_50_ values determined to inhibit NPC-TW01 cell growth *in vitro* (Fig. 4A). Therefore, the NPC-TW01 bearing mice were treated with i.p. injection of 0.5 mg/kg of squamostatin A every three days. Paclitaxel (10 mg/kg, weekly injection) was used as a positive control. The results clearly showed that squamostatin A significantly retarded NPC-TW01 cell growth after 5 weeks of treatment compared to paclitaxel (Fig. 4B). Overall tumor volume was stable between weeks 6 and 13, whereas tumor continued growth slowly in the paclitaxel-treated group (Fig. 4B). Except for a slight body weight increase in the squamostatin A-treated animals, no differences were found among non-drug-treated control animals and squamostatin A-treated animals in food consumption and clinical signs of toxicity.

## Discussion

Recent studies suggest that solid tumor cells exhibit increased survival due to ESR caused by an unfavorable microenvironment such as hypoxia, free-radical insult, pH change and misfolded mutated proteins. However, prolonged cellular stress leads to chronic ESR and ultimately cell death. However, normal cells are not subjected to ESR and the UPR pathway is inactive; therefore, targeting ER stress and/or the UPR represents a novel tumor treatment with limited effects on healthy cells^47^. Recently, a number of drugs targeting the ESR have been tested and have shown promising results against tumor proliferation *in vitro* and *in vivo*^31^,^32^,^34^.

NPC, rare cancer in Western society, is extremely common in South-East Asia. In Taiwan, NPC is the tenth leading cause of mortality in male cancer patients and the most common among males between 35 and 50 years of age. As a result, the impact of NPC on family and social stability is quite devastating. Clinically, distant metastases are found in the majority of patients diagnosed with NPC. Although pre-radiation chemotherapy may enhance the five-year survival rates of NPC patients; however, the development of drug resistance often results in failed treatments. Therefore, novel therapeutic drugs and protocols are urgently needed to overcome drug-resistance in NPC patients.

To search for agents that could effectively treat NPC, hundreds of compounds were evaluated for antitumor efficacy against two human NPC cell lines in our laboratory. These screening results identified several ACG compounds that effectively inhibit the growth of NPC cells with IC_50_ values in the nanomolar range. In particular, three THF-ACGs were able to overcome methotrexate-resistance. Moreover, all three THF-ACGs induced ESR, cytosolic and mitochondrial Ca^2+^ accumulation, mitochondrial damage, G_2_/M cell cycle-arrest, caspase activation and apoptotic cell death.

Squamostatin shows a great impact on cellular function, from inhibiting SOCE activation, inducing mitochondrial damage and ER stress. To the best of our knowledge, we are the first to clearly demonstrate the activation of ESR by the THF-ACG-induced release of Ca^2+^ from the ER into the cytosol. In the continued presence of ACG, the protective components of the ESR were unable to restore proper calcium homeostasis and therefore triggered the proapoptotic signaling pathway to initiate tumor cell death. Our studies also clearly demonstrated that squamostatin A treatment increased the expression or activity of components of the ESR pathway, including up-regulating transcription and translation level of GRP78/BiP, CHOP/GADD153 (Fig. 1A,B) and the phosphorylation of IRE-1〈, PERK and c-Jun (Fig. 1B). In addition, the role squamostatin A treatment in the intrinsic apoptotic pathway became evident as determined by the activation of caspase-9/-3 and Bax expression (Fig. 1D,E), which is consistent with the current model of ESR-induced cell death. Moreover, GADD34 knockout and caspase-12 inhibition experiments further suggested that squamostatin A-induced ER stress and cell death were correlative.

Contrary to previous reports, our results demonstrated that ESR induction by acetogenins was independent of extracellular Ca^2+^ (Fig. 2); however, the presence of extracellular Ca^2+^ promoted the apoptotic process (Fig. 1C). The difference between our results and the results published by Liaw *et al*. may reflect differences in cancer cell models employed. Although the target molecule(s) responsible for the induction of NPC-specific ESR by THF-ACGs was not identified in this study, further experiments to identify these molecules are actively underway. Furthermore, according to the number and stereochemistry of THF, ACG have been classified into non-THF, mono-THF, adjacent bis-THF, non-adjacent bis-THF, and tri-THF acetogenins^7^,^48^. However, the knowledge of essential pharmacore structure and structure-activity-relationship of acetogenins still need to be exploited. In order to identify new compound with better tumor growth inhibitory activity with high tumor selectivity, several newly synthesized ACGs derivatives including different number of THFs, various space between the THF groups and new substitutional groups had been synthesized and tested in our laboratory now.

For the past 30 years, the lack of clarity regarding the antiproliferative mechanisms of ACGs has hindered their development and use in the clinical settings. By improving our understanding of the antiproliferative mechanism of ACGs, we have brought these potent anticancer agents one-step closer to clinical use. In particular, these compounds may become much needed alternative treatments for drug-resistant nasopharyngeal malignancies.

## Materials and Methods

### Isolation of THF-ACGs

Squamostatin A, squamocin M and corossolone were isolated from the seeds of *Annona squamosa* L. (Annonaceae), and the plant materials were identified and authenticated by Dr. Tran Huy Thai (Institute of Ecology and Biological Resources, Vietnamese Academy of Science and Technology). The structure of each compound was identified by 1D-, 2D-NMR and HR-ESI-MS, and their spectroscopic data were compared to those reported in the literature^49^,^50^,^51^.

### Antibodies and Reagents

Primary antibodies against Grp78, GADD34, CHOP, p-Ire-1 α, p-PERK-1 and p-c-Jun were purchased from Cell Signaling Technology (Danvers, MA); Bax, bcl-2, bcl-x, actin and horseradish peroxidase-conjugated secondary antibodies were purchased from Santa Cruz Biotechnology (Santa Cruz, CA). CaspGLOW Fluorescein Active Caspase Staining Kits were purchased from Biovision (Milpitas, CA). Cell culture media were obtained from Hyclone (South Logan, UT). DiOC_6_, Fluo-3 and Rhod-2 were purchased from Invitrogen (New York, USA). Western blot chemiluminescence reagents were purchased from Millipore (Boston, MA). The lentiviral siRNAs, shGADD34 (TRCN03041) and shGFP (TRCN 072178) were purchased from National RNAi Core Facility (Taipei, Taiwan) and DNA sequence is shown in Supplemental Table 3. All other chemicals were obtained from Bio-Rad (Richmond, CA), USB (Darmstadt, Germany) or Sigma Chemical (St. Louis, MO) and were the molecular biologic grade or higher. The use of the toxic chemicals followed the rules of Toxic Chemical Substances Control Act of Taiwan. All the following experiments also followed the guidelines of Good Laboratory Practice of Taiwan.

### Cell Lines

Human leukemia (Jurkat), non–small cell lung carcinoma (NCI-H226), nasal pharyngeal carcinoma (HONE-1), oral carcinoma (FaDu), hepatocellular carcinoma (Hep 3B) and human normal fibroblasts (Detroit 551), were obtained from the American Type Culture Collection (Rockville, MD). A nasopharyngeal carcinoma (NPC-TW01) cell line was purchased from the Taiwan Food Industry Research and Development Institute (Hsinchu, Taiwan). All of the tumor cell lines were maintained in MEM, RPMI 1640 or DMEM supplied with 10% fetal bovine serum at 37 °C in a humidified atmosphere of 5% CO_2_/95% air in the presence of antibiotics.

### Growth inhibition assay (MTT assay)

The colorimetric assay for cellular growth and survival was performed as described by Hansen *et al*., with slight modifications^52^. Pre-determinate numbers of cells were seeded in a 96-well microplate and designated compounds at various concentrations were added for the indicated time period. MTT containing solution was added after compound treatment and conversion of MTT to formazan by metabolically viable cells was measured by absorbance at 490 nm in a 96-well microtiter plate reader. The percentage of conversion by mock-treated control cells was used to evaluate the effect of the chemicals on cell growth and to determine the IC_50_ concentration.

### Quantitative real-time PCR analysis

Total RNA was isolated from NPC-TW01 cells using the Trizol reagent and cDNA was generated by Rexert aid^TM^ M-Mulr Reverse Transcriptase kit. Q-PCR was performed using Applied Biosystems StepOne™ system and the amplification program was as follows: enzyme activation at 94 °C for 10 mins, followed by 40 cycles of three-step PCR with denaturation at 94 °C for 30 seconds, annealing at 60 °C for 30 seconds and extension at 72 °C for 30 seconds. GAPDH was used as an endogenous control to correct for variation in RNA loading and the primer set sequence for the reaction is listed in Supplemental Table 2. Relative quantitation was performed using the comparative C_T_ (ΔΔC_T_) method^53^.

### Western blot analysis

Exponentially growth NPC cells were treated with various concentrations of THF-ACGs for the indicated time and cells were lysed by SDS-containing lysis buffer. An equal amount of lysate protein was separated by SDS-PAGE gels, transferred to PVDF membranes and protein expression level was detected by Enhanced chemiluminescence following the manufacturer’s protocol. Images were taken on an LAS-4000 (Fuji Film, Japan).

### Flow cytometry

To measure the THF-ACGs effect on the cell cycle distribution, the THF-ACGs-treated cells were collected, fixed with ice-cold alcohol, treated with RNase and stained with propidium iodide. The DNA content of each sample was evaluated by FACScan flow cytometer and ratio of cell cycle phases were determined by ModFit software (Verity Software House Inc., Topsham, ME).

To measure the change of mitochondrial potential transition (MPT), THF-ACGs treated cells were labeled with 100 nM DiOC_6_, harvested and suspended in PBS. The intensity of DiOC_6_ in cells was measured using FACScan flow cytometer.

THF-ACGs induced apoptotic phenomena were monitored by The Annexin V-FITC Apoptosis Detection Kit II (BD Biosciences Pharmingen) according to the manufacturer’s instructions. Briefly, THF-ACGs-treated or control cells were collected and resuspended in 400 μL 1 × binding buffer at a concentration of ~1 × 10^6^ cells/ml, and 5 μL of purified recombinant Annexin V and PI reagent were added. After incubation at room temperature for 15 min in the dark, the intensity of Annexin V and PI was measured by flow cytometer immediately and results were analyzed using De Novo software (MultiCycle AV Plug-in for FCS Express).

To determine the activity of caspases-3, -8, -9 and -12 on squamostatin-A–treated cells, the CaspGLOW Fluorescein Active Caspase Staining Kit was used to measure the cleavage of specific fluorogenic peptide substrates, according to the manufacturer’s instructions and results were analyzed using De Novo software.

The cytosolic and mitochondrial Ca^2+^ concentrations were measured using fluo-3 and Rhod-2, respectively. The intensity of de-esterification of intracellular AM esters in the THF-ACGs treated or control tumor cells were measured after 30 mins of incubation. For each analysis, 10,000 events were recorded and results were analyzed using De Novo software.

### Single cell [Ca^2+^]_i_ measurement

Intracellular Ca^2+^ was measured at 37 °C with the Fura-2 fluorescence ratio method on a single-cell fluorimeter. In brief, cells attached on glass-bottom dishes were loaded with 2 μM Fura-2 Fura-2/acetoxymethyl ester (Fura-2/AM) in serum-free culture medium at 37 °C for 30 min. Cells were then washed three times with PBS. The dish was then placed on the stage of an Olympus IX71 inverted microscope equipped with a xenon illumination system and an IMAGO CCD camera (TILL Photonics). The excitation wavelength was alternated between 340 nm (*I*_340_) and 380 nm (*I*_380_) using the Polychrome IV monochromator (TILL Photonics). The fluorescence intensity of excitation at IS 510 nm was monitored to calculate the intracellular Ca^2+^ levels by TILLvisION 4.0 program (Till Photonics). ER Ca^2+^ release was induced with 2 μM TG for 10 minutes in the absence of extracellular Ca^2+^, followed by the activation of SOCE with the addition of 2 mM Ca^2+^.

### Caspase Inhibition Assay

For caspase inhibition experiments, cell-permeable and irreversible inhibitors of caspase-12 were added to the medium one hour before the administration of squamostatin A. Cell survival was determined using the MTT assay to evaluate the effects of the test compound on cell growth, as described previously.

### GADD34 Knockdown NPC-TW01

To generate transient transfected GADD34 knockdown cells, 10 μg of shGADD34 lentiviral siRNAs were transfected into NPC-TW01 cells by lipofectamine following commercial manual. Briefly, cells were incubated with lipofectamine in the present of single or multiple shGADD 34 lentiviral plasmid. After 24 hours incubation, cells were pooled and expanded for further experiments. The shGFP-transfected NPC-TW01 cells were used as a control. Compared the knock-out efficiency of all the shGADD34-transfected cell, the No. 1 + 2 + 3 + 4 transfected cells were chosen for following experiments (Supplemental Fig. 2).

### Animals

For the squamostatin A pharmacokinetic (PK) studies, BALB/c mice 6 to 8 weeks of age, were fasted for 15 hours before drug administration, water was supplied ad libitum and the food was only supplied 3 hours after dosing. The mice received 0.5 or 1 mg/kg of squamostatin A by i.p. administration. For each dosage, the mice were randomly divided into two groups (5 in each group). At various time points post-dosing, 100 μL of blood was collected via cardiac puncture from the alternative groups. All serum obtained was stored at −20 °C for further analysis. To determinate the amount of squamostatin A in the serum, the serum was acidified with 0.1 N HCl, partitioned with ethyl acetate. The ethyl acetate was removed under N_2_ gas and reconstituted with 50 *μ*L of the mobile phase. Twenty *μ*L of sample was subjected to LC/MS/MS analysis. The concentration of squamostatin A in the serum was determined using a standard squamostatin A. Values represent the mean (±SD) for five animals per group.

For the antitumor xenograft experiments**, s**pecific pathogen–free male athymic BALB/c nude mice, 6 to 8 weeks of age, were inoculated with 1 × 10^6^ of exponential growth viable NPC-TW01 cells at the right flank of mice and mice were randomly divided into three groups (8 in each group). Tumor-implanted mice were treated i.p. with vehicle (5% DMSO/10% cremophor/85% saline) or 0.5 mg/kg squamostatin A every three days. Paclitaxel (10 mg/kg, once a week) was used as a positive control. Tumor size was measured every three days with a caliper, and tumor volume was calculated by the following formula: V = (1/2) × (larger diameter)^2^ × (smaller diameter). At the end of the experiments, the animals were euthanized by carbon dioxide inhalation, followed by cervical dislocation. The animal experiments were supervised and approved by the Institutional Animal Care and Use Committee (IACUC) of China Medical University (Taichung, Taiwan). All animal studies were performed in accordance with the Animal Protection Act of Taiwan.

### Statistical Analysis

All assays were carried out in triplicate. The data are expressed as the mean with standard deviation (SD). Student’s *t*-tests were used to compare the mean of each group with that of the control group. A *p*-value of p < 0.05 (*) or p < 0.01(**) was considered statistically significant.

## Additional Information

**How to cite this article**: Juang, S.-H. *et al*. Mechanistic Study of Tetrahydrofuran- acetogenins In Triggering Endoplasmic Reticulum Stress Response-apotoposis in Human Nasopharyngeal Carcinoma. *Sci. Rep.* **6**, 39251; doi: 10.1038/srep39251 (2016).

**Publisher's note:** Springer Nature remains neutral with regard to jurisdictional claims in published maps and institutional affiliations.

## Supplementary Material

Supplementary Data

## Figures and Tables

**Table 1:**
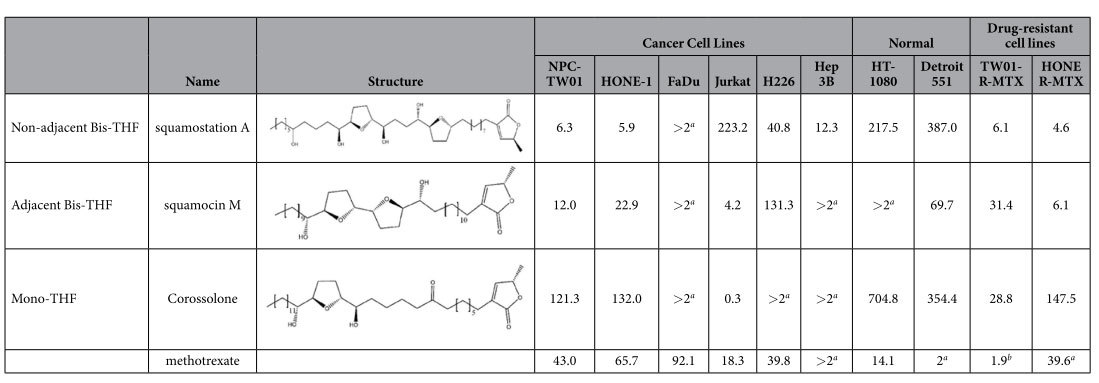
Growth inhibitory activity of THF-ACGs against various human cell lines, IC_50_ (nM).

Cells were treated with various concentrations of acetogenins or methotrexate (an anticancer drug as a positive control) for three generations. Cell growth was determined using an MTT colorimetric assay. Representative data from three independent experiments performed in quadruplicate are shown. ^*a*^IC_50_ (μM),^*b*^IC_50_ (mM).

**Table 2:** Effects of THF-ACGs on cell cycle distribution in NPC-TW01 cells.

Treatment	Cell cycle distribution (%)		
	G_0_/G_1_	S	G_2_/M
Control	61.4	24.9	13.8
squamostatin A	43.7	24.4	32.0
squamocin M	40.6	25.1	34.3
corossolone	40.9	28.6	30.5

NPC-TW01 cells were treated with the IC_50_ concentration of THF-ACGs for 24 hours and analyzed for DNA content by PI-stained using a flow cytometer. The Control treatment indicates that cells were treated with 0.1% dimethyl sulfoxide.

**Table 3:** Effects of THF-ACGs on the induction of cell apoptosis in NPC-TW01 cells.

Treatment	Annexin-V positive (%)		
	E	L	E + L
Control	1.4	1.9	3.3
squamostatin A	15.9	3.0	18.9
squamocin M	5.4	10.6	16.0
corossolone	4.9	7.4	14.3

NPC-TW01 cells were treated with the IC_50_ concentration of THF-ACGs for 36 hours, incubated with Annexin-V and PI and subjected to flow cytometric analysis. The Control treatment indicates that cells were treated with 0.1% dimethyl sulfoxide. E: early phase apoptosis; L: late phase apoptosis.

**Table 4:** Effects of THF-ACGs on the intracellular calcium concentrations (cytosolic and mitochondria) and mitochondrial membrane potential in NPC-TW01 cells.

Treatment	GEO mean value					
	Ca^2+^					
	Cytosol (Fluo-3)		Mitochondria (Rho-2)		Mitochondrial membrane potential	
	Ctrl	Treated	Ctrl	Treated	Ctrl	Treated
quamostatin A	62.2	93.5 (↑50%)	198.0	260.0 (↑31%)	1248	787 (↓37%)
squamocin M	82.7	179.3 (↑116%)	116.2	152.1 (↑30%)	1057	710 (↓33%)
corossolone	73.5	149.2 (↑123%)	116	149.2 (↑29%)	1059	777 (↓27%)

NPC-TW01 cells were treated with the IC_50_ concentration of THF-ACGS for 24 hours, and the cytosolic and mitochondria calcium concentrations of the cells were measured by the calcium indicator dyes, Fluo-3 and Rhod-2, respectively (*left part of table*). In addition, the reduction of mitochondrial transmembrane potential by THF-ACGs was measured with DiOC_6_ staining and flow cytometry (*right part of table*). The Control treatment (Ctrl) indicates that cells were treated with 0.1% dimethyl sulfoxide.
