# Supplementary material for: Mechanistic Study of Tetrahydrofuran- acetogenins In Triggering Endoplasmic Reticulum Stress Response-apotoposis in Human Nasopharyngeal Carcinoma
Source: Sci Rep. 2016 Dec 21;6:39251. doi: 10.1038/srep39251 (PMC5175284; doi:10.1038/srep39251)
Supplement: Supplementary Data [file srep39251-s1.pdf]

Supplementary Information

Mechanistic Study of Tetrahydrofuran- acetogenins In Triggering Endoplasmic Reticulum Stress Response-apotoposis in Human Nasopharyngeal Carcinoma

Shin-Hun Juang[1,2+], Chang-Ying Chiang[3+], Fong-Pin Liang[1], Hsiu-Hui Chan[4], Jai-Sing Yang[2], Shih-Hao Wang[3],Yu-Chin Lin[3]Ping-Chung Kuo[4], Meng-Ru Shen[5],Tran Dinh Thang[6], Bui Thi Minh Nguyet[6], Sheng-Chu Kuo[3], Tian-Shung Wu[4,*]

Supplementary figures

(A)

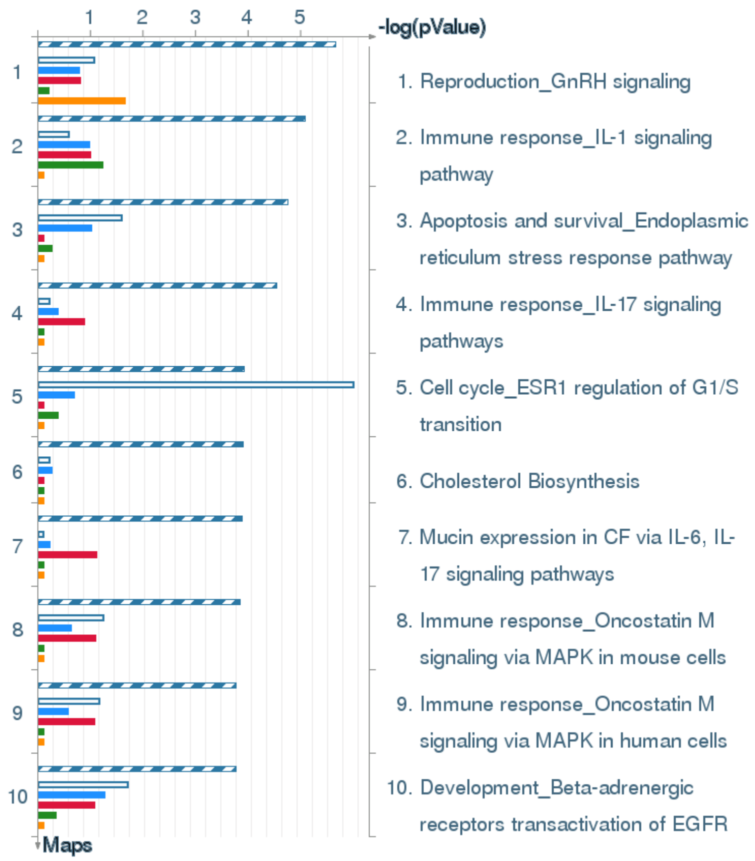

(B)

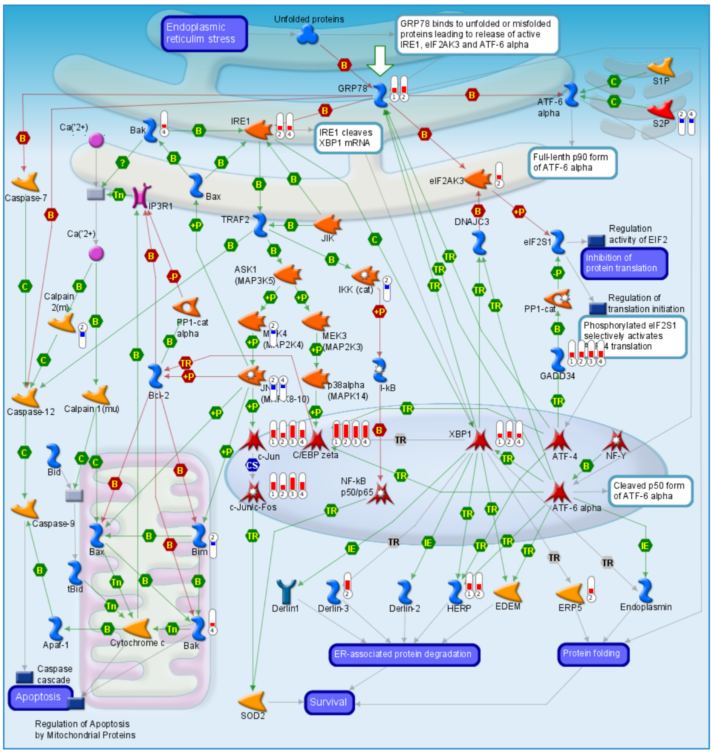

Supplemental Figure 1. (A) Squamostatin A affected gene expression in NPC-TW01 cells. Cells were exposed to 6 μM and 12 μM squamostatin A for 24 and 36 hours were harvested, and total RNA was isolated for DNA microarray analysis. Microarray results showed that, compared with the untreated control cells, 84 genes were up-regulated and 85 genes were down-regulated. The microarray analytic data were further subjected to comprehensive pathway analysis by canonical pathway maps, which represent a set of approximately 650 signaling and metabolic maps covering human biology (signaling and metabolism). All maps were drawn from scratch by GeneGo annotators and manually curated and edited. Experimental data are visualized on the maps by blue (down-regulation) and red (up-regulation) histograms. The height of the histogram corresponds to the relative expression value for a particular gene. (B) The scored map was based on the enrichment distribution sorted by the 'common' set. Experimental data from all files is linked to and visualized on the maps as thermometer-like figures. Upward thermometers are red and indicate up-regulated signals, and downward thermometers are blue and indicate down-regulated gene expression levels.

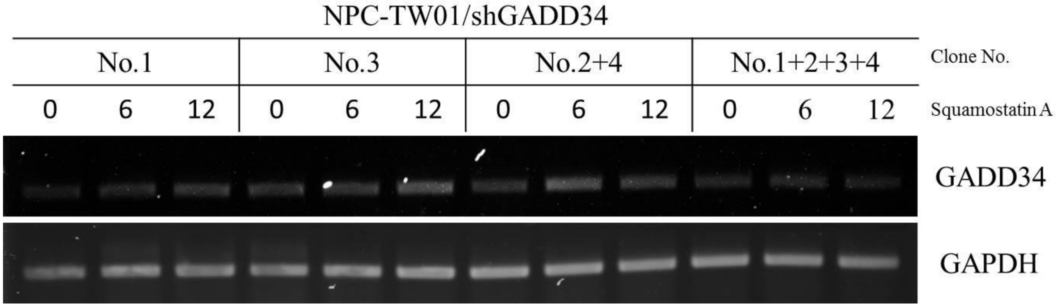

Supplemental Figure 2. The GADD34 mRNA level of transient transfected NPC-TW01 cells. The transient GADD34 knockdown cells were transfected total 10 μg of verity shGADD34 lentiviral siRNA clones by lipofectamine method. Twenty-four hours after lipofectamine was removed, the cells were treated with different concentration of squamostatin A for 24 hours, and total mRNA was collected by TRIzol treatment.

Supplementary tables

Supplemental Table 1. Growth inhibitory activity of THF-ACGs against various oral cancer lines.

Cell lines	Types	IC_{50}		
		squamostatin A	squamocin M	corossolone
NPC-TW01	Human nasopharyngeal carcinoma cell line	6.30 nM	1.1 nM	31.3 nM
HONE-1	Human nasopharyngeal carcinoma cell line	5.87 nM	22.9 nM	132.0 nM
FaDu	Human pharynx squamous cell carcinoma	>2 μM	>2 μM	>2 μM
OECM-1	Human oral epidermoid carcinoma cell line	>2 μM	>2 μM	>2 μM
SCC-15	Human tongue squamous cell carcinoma	>2 μM	>2 μM	>2 μM
SCC-25	Human tongue squamous cell carcinoma	>2 μM	>2 μM	>2 μM

Cell growth was determined by an MTT colorimetric assay. Representative data from three independent experiments performed in quadruplicate are shown.

Supplemental Table 2. Q-PCR primer set

Primer set		Sequence(5’→3’)
Grp78	F	CTGTCCAGGCTGGTGTGCTCT
	R	CTTGGTAGGCACCACTGTGTTC
GADD34	F	TCCGACTGCAAAGGCGGCTCA
	R	CAGCCAGGAAATGGACAGTGAC
GADD153	F	GGTATGAGGACCTGCAAGAGGT
	R	CTTGTGACCTCTGCTGGTTCTG
GAPDH	F	GTCTCCTCTGACTTCAACAGCG
	R	ACCACCCTGTTGCTGTAGCCAA

Supplemental Table 3. shRNA clones information

No.	Gene	Vector	Region	Target Sequence
1	*sh*GADD34	pLKO.1	CDS	CGAGAAGGTCACTGTCCATTT
2	*sh*GADD34	pLKO.1	CDS	GTGGATAGTGAGGATAAGGAA
3	*sh*GADD34	pLKO.1	CDS	GACACTGCAAGGTTCTGATAA
4	*sh*GADD34	pLKO.1	CDS	GATGAGGACAGTGATACAGGA
5	*sh*GFP	pLKO.1	3UTR	CAACAGCCACAACGTCTATAT

The shRNA reagents were obtained from the National RNAi Core Facility located at the Institute of Molecular Biology/Genomic Research Center, Academia Sinica, Taiwan, ROC. The single or multiple *sh*GADD34 lentiviral siRNAs were transfected into NPC-TW01 cells by lipofectamine. After 24 hours incubation, transfected cells were pooled and expanded for further experiments. The *sh*GFP (TRCN 072178) transfected NPC-TW01 cells were used as a control.
